# Outcomes of highly active antiretroviral therapy in the context of universal access to healthcare: the U.S. Military HIV Natural History Study

**DOI:** 10.1186/1742-6405-7-14

**Published:** 2010-05-27

**Authors:** Vincent C Marconi, Greg A Grandits, Amy C Weintrob, Helen Chun, Michael L Landrum, Anuradha Ganesan, Jason F Okulicz, Nancy Crum-Cianflone, Robert J O'Connell, Alan Lifson, Glenn W Wortmann, Brian K Agan, the Infectious Disease Clinical Research Program HIV Working Group (IDCRP)

**Affiliations:** 1 Infectious Disease Clinical Research Program, Uniformed Services University of the Health Sciences, Bethesda, MD, USA; 2 Infectious Disease Service, San Antonio Military Medical Center, San Antonio TX, USA; 3 Division of Biostatistics, University of Minnesota, Minneapolis, MN, USA; 4 Infectious Disease Service, Walter Reed Army Medical Center, Washington, DC, USA; 5 Infectious Disease Clinic, Naval Medical Center San Diego, San Diego, CA, USA; 6 Infectious Disease Clinic, National Naval Medical Center, Bethesda, MD, USA; 7 Walter Reed Army Institute of Research, Rockville, MD, USA; 8 Emory University School of Medicine, Atlanta, GA, USA

## Abstract

**Background:**

To examine the outcomes of highly-active antiretroviral therapy (HAART) for individuals with free access to healthcare, we evaluated 2327 patients in a cohort study composed of military personnel and beneficiaries with HIV infection who initiated HAART from 1996 to the end of 2007.

**Methods:**

Outcomes analyzed were virologic suppression (VS) and failure (VF), CD4 count changes, AIDS and death. VF was defined as never suppressing or having at least one rebound event. Multivariate (MV) analyses stratified by the HAART initiation year (before or after 2000) were performed to identify risk factors associated with these outcomes.

**Results:**

Among patients who started HAART after 2000, 81% had VS at 1 year (N = 1,759), 85% at 5 years (N = 1,061), and 82% at 8 years (N = 735). Five years post-HAART, the median CD4 increase was 247 cells/ml and 34% experienced VF. AIDS and mortality rates at 5 years were 2% and 0.3%, respectively. In a MV model adjusted for known risk factors associated with treatment response, being on active duty (versus retired) at HAART initiation was associated with a decreased risk of AIDS (HR = 0.6, 95% CI 0.4-1.0) and mortality (0.6, 0.3-0.9), an increased probability of CD4 increase ≥ 50% (1.2, 1.0-1.4), but was not significant for VF.

**Conclusions:**

In this observational cohort, VS rates approach those described in clinical trials. Initiating HAART on active duty was associated with even better outcomes. These findings support the notion that free access to healthcare likely improves the response to HAART thereby reducing HIV-related morbidity and mortality.

## Background

Despite substantial progress since the introduction of highly-active antiretroviral therapy (HAART) [1-4], maintaining virologic suppression is predominantly challenged by suboptimal antiretroviral (ARV) adherence. Studies have shown that difficulty with adherence is usually associated with (1) significant barriers to care, (2) ARV intolerability and (3) individual factors such as education, treatment fatigue, and the psychosocial context of the patient [5-7].

We sought to examine a large, multicenter cohort composed of military personnel and beneficiaries with HIV infection followed since diagnosis in order to illustrate the HAART outcomes for patients within a free-access healthcare system in the United States. The U.S. military medical system provides comprehensive HIV education, care and treatment, including the provision of ARVs and regular visits with HIV clinicians at medical treatment facilities (MTF), at no cost to the patient. Mandatory periodic HIV screening according to Department of Defense (DoD) policy [8] allows treatment initiation to be considered at an early stage of infection. Active duty personnel are required to attend the MTF at least twice yearly for formal medical evaluations. Following retirement from active duty, or separation for medical disability, all individuals retain health benefits and may continue participation in the cohort study while receiving their primary HIV care either within or outside of the military healthcare system.

Aside from the advantages afforded by the medical system, there are aspects of this cohort that allow for a unique perspective on HIV treatment response. The military population from which these patients are derived consists of highly motivated and disciplined individuals who possess either a minimum of a high school equivalent education (enlisted) or an undergraduate college degree (officers) and maintain rigorous physical standards [9-11]. As a consequence of periodic random drug screening, the reported rate of injection drug use (IDU) in this population is less than one percent [12]. Thus, many of the factors which typically hinder the clinical response to HAART in most North American cohorts [13-15], such as IDU, homelessness and unemployment, are minimized or eliminated in the military setting. Additionally, the cohort is racially balanced and geographically diverse reflecting the distribution of individuals with HIV in the U.S.[16]. As a separate aim, this cohort provided an opportunity to examine the relationship between demographic (e.g. race/ethnicity) and clinical factors (hepatitis B, prior STI, etc.) with outcomes after HAART in a U.S. population with fewer confounders related to access to care and IDU.

## Methods

### Study Participants

The U.S. Military HIV Natural History Study (NHS) is a prospective multicenter observational study of HIV-infected active duty military personnel and other beneficiaries (spouses, dependents, and retired military personnel) from the Army, Navy/Marines and Air Force. All participants provided written informed consent. The cohort characteristics have been previously described [17]. Patients were included in this analysis if they were enrolled in the NHS and initiated HAART at any time from 1996 until December 31, 2007 with data collected through July 1, 2008. The NHS has been approved by the Institutional Review Board of each participating center.

### Definitions

Seroconverters (SC) were defined as patients having a documented HIV seronegative date prior to the first positive HIV date. The estimated date of seroconversion for SC was defined as the midpoint between the two dates. All CD4 count and VL measurements were done as part of routine clinical care. The clinically-approved methodology for this testing varied by site and over time. Sexually transmitted infections (STIs) were defined as having a documented clinical history of gonorrhea, chlamydia, syphilis or herpes simplex at any time prior to initiation of HAART. Chronic hepatitis B co-infection was defined as having at least two positive hepatitis B surface antigen tests at least 6 months apart. Hepatitis C virus (HCV) co-infection was defined as having at least one positive HCV antibody test. ARV use referred to any antiretroviral therapy not meeting the NHS definition of HAART [17]. HAART initiation was the date when HAART was first prescribed. AIDS-defining illnesses were defined using the 1993 CDC classification but did not include CD4 count < 200 as an endpoint [18].

### Statistical Analysis

Outcomes were described for all patients and separately for those initiating HAART from 1996-1999 (early HAART era, EHE) and for those starting HAART in 2000-2007 (late HAART era, LHE). Virologic outcomes and CD4 cell count response were described at 6-month intervals through 8 years after the initiation of HAART. Due to differing lengths of follow-up after HAART initiation, the sample size was 1063 (46%) at 5 years and 735 (32%) at 8 years. CD4 and viral load (VL) at HAART were the last recorded value up to 6-months before HAART. Six-month follow-up values where those recorded closest to the 6-month interval after HAART initiation (within a window of ± 3 months). Patients with missing laboratory values for a given time point were excluded from analyses at that time point. Virologic suppression (VS) was defined as an undetectable viral load (< 400 copies/mL). Virologic failure (VF) was defined as 2 consecutive VL detectable after VS (virologic rebound) or never achieving VS (never suppressed). Always suppressed was defined as having all measured VL undetectable for the entire period beginning 6 months after HAART initiation. CD4 count outcomes were expressed as the group mean and the mean increase after HAART initiation at a given time point. The percentage of patients who experienced at least a 30% or 50% CD4 count increase from HAART initiation was also determined. Switches and discontinuations of ARVs were not counted as failures.

Kaplan Meier (KM) life-table methods were used to estimate the cumulative rate of VF, CD4 increase of 50%, AIDS-defining conditions, and all-cause mortality. Patients without the event of interest were censored at the last recorded visit. For time-to-VF, patients never suppressed were considered to have failed at time zero. Stratified Cox-regression (by HAART initiation era and medical center) was used to determine the association of relevant covariates with these same outcomes. Baseline covariates used in the model were those found to be associated (p < 0.1) in univariate analyses as well as those shown to be risk factors in the literature.

## Results

### Baseline Characteristics

Characteristics for patients who initiated HAART overall and by HAART initiation era are shown in Table 1. A total of 2,327 patients initiated HAART; 1,631 during the EHE and 696 during the LHE. Average follow-up after initiation of HAART was 6.2 years for all patients, 7.4 years in the EHE and 3.4 years in the LHE. The mean age at HAART start was 35 years overall and 9.5% were women. The race/ethnicity distribution was equally divided between African and European Americans (44% each); 8% were Hispanic and 4% were of other race/ethnicities. Overall, 213 (9.9%) were commissioned or warrant officers at study enrollment; 56% were active duty at time of HAART. The mean CD4 level at HAART start was 343 cells/mL and was similar in both eras. Patients in the LHE were more likely to be active duty, have a shorter duration between HIV diagnosis and HAART initiation, and less likely to have an AIDS-defining illness prior to HAART initiation, than those in the EHE.

**Table 1:** Baseline Factors for Patients Initiating HAART in the Natural History Study

Characteristic	Total (n = 2327)	Early Initiation Era (n = 1631)	Late Initiation Era (n = 696)	**P value**^**b**^
**Demographics**				
Age at HIV Diagnosis, years	30.1 ± 8.1	29.8 ± 7.9	30.8 ± 8.6	0.007
Age at HAART initiation, years	34.7 ± 8.6	35.0 ± 8.2	34.1 ± 9.5	0.025
Female	221 (9.5%)	169 (10.4%)	52 (7.5%)	0.029
Race/ethnicity				0.188
European American	1024 (44.0%)	730 (44.8%)	294 (42.2%)	
African American	1021 (43.9%)	717 (44.0%)	304 (43.7%)	
Hispanic	194 (8.3%)	130 (8.0%)	64 (9.2%)	
Other	88 (3.8%)	54 (3.3%)	34 (4.9%)	
Rank of Officer/Warrant at study enrollment	213 (9.9%)	143 (9.4%)	70 (10.1%)	0.606
Active Duty at HAART initiation	1293 (55.6%)	773 (47.4%)	520 (74.7%)	<0.001
**Medical History (prior to HAART Initiation)**				
Year of HIV Diagnosis	1994 ± 5.9	1992 ± 4.2	2000 ± 5.1	<0.001
HIV Diagnosis to HAART initiation, months	44.2 (5.7 - 95.2)	60.9 (16.9 - 103.8)	10.1 (2.0 - 45.5)	<0.001
Nadir CD4^+ ^to HAART initiation, months	3.3 (0.4 - 16.3)	6.5 (0.7 - 19.4)	0.8 (0.2 - 3.7)	<0.001
Seroconverters (SC)^a^	1691 (72.7%)	1106 (67.8%)	585 (84.1%)	<0.001
Estimated date of SC to HIV Diagnosis, months	8.1 (5.0 - 13.7)	8.4 (5.3 - 14.4)	7.4 (5.0 - 13.7)	0.010
Viral Load at HAART initiation, log_10 _copies/mL	4.3 ± 1.0	4.3 ± 1.0	4.4 ± 0.9	<0.001
CD4^+ ^at HIV Diagnosis, cells/mL	499.7 ± 248.0	524.0 ± 252.1	448.0 ± 231.0	<0.001
CD4^+ ^nadir, cells/mL	283.2 ± 174.0	276.4 ± 183.3	299.6 ± 148.1	0.005
CD4^+ ^at HAART Initiation, cells/mL	342.8 ± 211.6	341.0 ± 223.4	346.6 ± 184.8	0.590
<200	459 (24.4%)	357 (28.0%)	102 (16.7%)	<0.001
200-349	581 (30.8%)	331 (26.0%)	250 (40.9%)	
350+	845 (44.8%)	586 (46.0%)	259 (42.4%)	
Prior AIDS-Defining Event	277 (11.9%)	231 (14.2%)	46 (6.6%)	<0.001
Chronic Hepatitis B co-infection	128 (6.1%)	110 (7.4%)	18 (2.9%)	<0.001
Hepatitis C co-infection	121 (6.1%)	99 (7.1%)	22 (3.6%)	0.002
Prior Sexually Transmitted Infections (STI)	1058 (45.5%)	839 (51.4%)	219 (31.5%)	<0.001
ARV Use (mono- or dual-therapy)	1224 (52.6%)	1121 (68.7%)	103 (14.8%)	<0.001
Hemoglobin, g/dL	14.1 ± 1.6	13.9 ± 1.6	14.4 ± 1.4	<0.001
ALT, μ/L	47.1 ± 53.7	48.2 ± 52.9	45.1 ± 55.2	0.336
Creatinine, mg/dL	1.0 ± 0.2	1.0 ± 0.2	1.0 ± 0.2	<0.001
**Initial HAART Regimen**				
Unboosted PI	1320 (56.7%)	1261 (77.3%)	59 (8.5%)	<0.001
Boosted PI	205 (8.8%)	121 (7.4%)	84 (12.1%)	
NNRTI	622 (26.7%)	169 (10.4%)	453 (65.1%)	
PI + NNRTI + NRTI	86 (3.7%)	71 (4.4%)	15 (2.2%)	
3 NRTI	94 (4.0%)	9 (0.6%)	85 (12.2%)	

Median (IQR) is presented for duration factors given in months

^a ^Percentage of patients who are known seroconverters

^b ^Late versus Early HAART initiation era

### Antiretroviral Use

**Figure 1:**
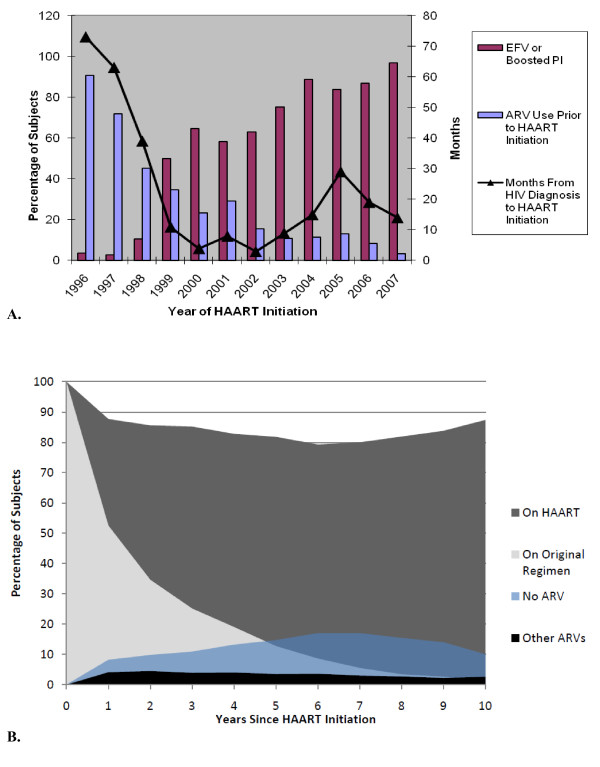
**HAART usage in the Natural History Study**. (A) Distribution of prior ARV use and first regimen type by year of HAART initiation with duration of HIV infection prior to HAART start for seroconverters. (B) Therapy changes over time. The declining percentage of patients remaining on the first HAART regimen results from complete discontinuation of or changes in therapy.

As expected, both prior ARV use and initial HAART regimen differed significantly (p < 0.001) between eras (Table 1, Figure 1A). Nearly 69% of patients in the EHE had prior ARV use compared to 15% in the LHE (p < 0.001). In the EHE, 85% used a PI-containing (77% unboosted) initial HAART regimen whereas in the LHE, 65% used an NNRTI-containing initial regimen (predominantly efavirenz). Of the 2,327 patients initiating their first HAART regimen, 557 (24%) remained on the same regimen for the entire duration of follow up; 53.5% were on their initial regimen at one-year (Figure 1B). At the end of follow-up, 84% were still on HAART. Of those still on HAART, 23% were on an unboosted PI, 23% were on a boosted-PI, and 27% were on a NNRTI. During the follow-up period, patients were on HAART an average of 93% of the time.

### VL, CD4 and Clinical Outcomes

The percentage of patients with VS (Table 2) was higher in the LHE compared to the EHE throughout follow-up (p < 0.001). One year after HAART initiation, 57% and 81% of patients with available viral loads had VS in the EHE and LHE, respectively. Restricting analyses to active duty patients, these percentages were slightly higher (64% and 84%, respectively). The percentage of patients with VS at 5 years was 59% and 85%, and at 8 years was 65% and 82% for the EHE and LHE, respectively. Analyses restricted to active duty patients showed nearly identical results at these time points. The cumulative percentage of patients who achieved an undetectable viral load ever within 5 years after HAART initiation was 93.2%. In a subset of patients where self-reported adherence was available within 15 months of HAART start (n = 133), over 94% reported ≥ 90% adherence. A cross-sectional assessment of adherence for all patients in the cohort on HAART (n = 1050) demonstrated over 90% reporting ≥ 90% adherence.

**Table 2:** Virologic and Immunologic Outcomes for patients initiating HAART using an Intention to Treat Analysis.

Outcome	1 year			5 years			8 years		
HAART Era	Total	Early	Late	Total	Early	Late	Total	Early	Late
Median (IQR) # of viral loads available per patient	4 (3-6)	4 (3-6)	5 (3-6)	17 (12-23)	18 (12-24)	15 (12-19)	26 (18-35)	26 (18-35)	20 (15-27)

Virologic Suppression (n)	1759	1216	543	1063	868	195	735	707	28
Suppressed^a^	1135 (64.5)	693 (57.0)^g^	442 (81.4)	674 (63.4)	508 (58.5)^g^	166 (85.1)	487 (66.3)	464 (65.6)	23 (82.1)
Always Suppressed^b^	864 (49.1)	478 (39.3)^g^	386 (71.1)	244 (23.0)	172 (19.8)^g^	72 (36.9)	112 (15.2)	104 (14.7)	8 (28.6)
Ever Suppressed^c^	1391 (79.1)	890 (73.2)	501 (92.3)	991 (93.2)	800 (92.2)	191 (97.9)	707 (96.2)	680 (96.2)	27 (96.4)

Virologic Failure^d^	629 (35.8)	525 (43.2)^g^	104 (19.2)	594 (55.9)	527 (60.7)^g^	67 (34.4)	496 (67.5)	482 (68.2)^g^	14 (50. 0)
Never Suppressed^e^	368 (20.9)	326 (26.8)^g^	42 (7.7)	72 (6.8)	68 (7.8)^g^	4 (2.1)	28 (3.8)	27 (3.8)	1 (3.6)
Rebound^f^	261 (14.8)	199 (16.4)^g^	62 (11.4)	522 (49.1)	459 (52.9)^g^	63 (32.3)	468 (63.7)	455 (64.4)	13 (46.4)

Mean CD4, cells/mL	488 ± 267	469 ± 268	530 ± 262	571 ± 306	562 ± 305	611 ± 307	556 ± 306	552 ± 301	657 ± 398
CD4 Change	143 ± 180	126 ± 171^g^	179 ± 193	220 ± 271	214 ± 270	247 ± 278	209 ± 288	206 ± 284	263 ± 362
CD4 Increase ≥ 30%	880 (60.0)	564 (56.9)	316 (66.5)	583 (66.9)	461 (65.3)	122 (73.5)	381 (62.5)	365 (62.6)	16 (59.3)
CD4 Increase ≥ 50%	665 (45.4)	418 (42.2)	247 (52.0)	489 (56.1)	385 (54.5)	104 (62.7)	331 (54.3)	318 (54.5)	13 (48.1)

Patients with missing lab values were excluded on that date

^a^Number (%) of patients at the given time point who have one undetectable viral load

^b^Number (%) of patients suppressed at 6-months and then at all visits through indicated time point

^c^Number (%) of patients having an undetectable viral load at least once through indicated time point

^d^Number (%) of patients at the given time point who have either had at least one episode of rebound or never suppressed

^e^Number (%) of patients never having an undetectable viral load

^f^Number (%) of patients ever having a rebound event (undetectable, then detectable + detectable)

^g^Significant difference comparing early versus late era (p < 0.05)

There were also significant differences between the eras in the percentage of patients who were always suppressed, never suppressed or had at least one virologic rebound event throughout the study period. At 1 year, 19% of patients in the LHE experienced VF (versus 43% EHE). For this same era at 5 and 8 years, there were 34% and 50% of LHE patients (versus 61% and 68% EHE), respectively. Similarly, the degree of immune reconstitution was greater in the LHE, despite similar CD4 levels at HAART start. In the first year, 52% of patients from the LHE had achieved a 50% gain in CD4 count. This increased to 63% of patients at 5 years.

**Figure 2:**
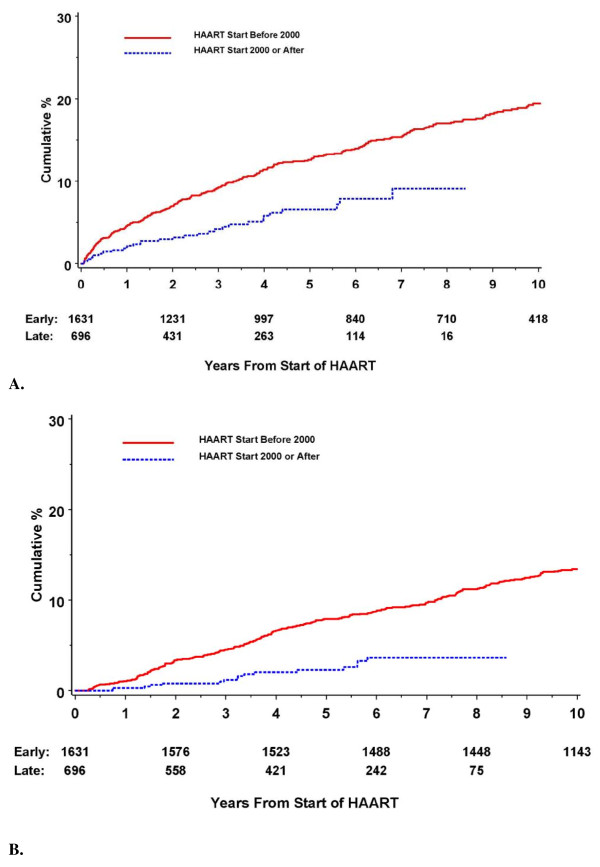
**KM curves for cumulative clinical outcomes for patients after HAART initiation stratified by HAART Era**. (A) First AIDS event (B) Mortality.

The rate of AIDS events and deaths were lower in the LHE compared to the EHE. At 1 year, the AIDS event rate (Figure 2) was 4.7% for patients in the EHE and 2.0% for patients in the LHE; the mortality rates were 1.0% and 0.3%, respectively. These rates remained low and the differences persisted throughout the study period.

### Predictors of Response to HAART

In a multivariate model (Table 3) stratified by HAART initiation era and MTF that included age, gender, ethnicity, active duty status, military rank, CD4 count, VL, duration of HIV infection, prior ARV use, initial HAART regimen, STIs, hepatitis B and C co-infection and Hgb, the factors significantly (p < 0.05) associated with VF were younger age at HAART initiation, African-American ethnicity, higher VL at HAART initiation, prior use of ARVs, and no prior history of STI. The factors significantly associated with achieving a CD4 cell gain of at least 50% were being on active duty at HAART start, lower CD4 count at HAART start, shorter duration of HIV infection, and no prior ARV use. Ethnicity nearly reached significance for this outcome. Risk factors associated with AIDS events after HAART were younger age, male gender, lower CD4 count, and prior AIDS events. Non-active duty status and duration of HIV infection showed a trend towards significance. Factors associated with higher mortality included non-active duty status, lower CD4 count at HAART initiation, higher VL at HAART initiation, HCV co-infection, and lower Hgb. No difference was seen when comparing PI to NNRTI use as the first regimen. Although patients on active duty had better clinical and immunologic outcomes as well as a higher likelihood of VS (data not shown), no difference was found with time to VF.

**Table 3:** Predictors of Time to Development of Outcomes after initiating HAART Using Multivariate Cox Proportional Hazards.

Risk Factor	Virologic Failure N = 1307	CD4 Response^a^ N = 1375	AIDS N = 1375	Mortality N = 1376
Age HAART start, 10 yrs	**0.8 (0.7-0.9), <0.001**	1.1 (1.0-1.2), 0.088	**0.7 (0.6-0.9), 0.019**	1.1 (0.8-1.4), 0.649
Gender, Women vs Men	1.2 (0.8-1.6), 0.381	1.0 (0.8-1.3), 0.987	**0.3 (0.1-1.0), 0.042**	0.6 (0.2-1.5), 0.240
Ethnicity, AA vs EA^b^	**1.2 (1.0-1.5), 0.015**	0.9 (0.8-1.0), 0.056	1.2 (0.8-1.8), 0.378	0.9 (0.6-1.4), 0.773
Active Duty, yes vs no	1.1 (0.9-1.4), 0.269	**1.2 (1.0-1.4), 0.036**	0.6 (0.4-1.0), 0.051	**0.6 (0.3-0.9), 0.021**
Rank, Enlisted vs Officer^b^	1.1 (0.8-1.4), 0.710	1.0 (0.8-1.3), 0.677	1.0 (0.5-1.8), 0.877	1.3 (0.7-2.6), 0.427
CD4 at initiation, 50 cells	1.0 (1.0-1.0), 0.765	**0.9 (0.8-0.9), <0.001**	**0.9 (0.8-0.9), <0.001**	**0.9 (0.8-1.0), 0.003**
VL at initiation, 1 log	**1.2 (1.1-1.3), <0.001**	1.1 (1.0-1.2), 0.074	1.1 (0.9-1.4), 0.352	**1.4 (1.1-1.8), 0.007**
Duration of HIV, 5 years	1.1 (1.0-1.23), 0.203	**0.9 (0.8-0.9), <0.006**	1.3 (1.0-1.8), 0.059	1.1 (0.8-1.5), 0.702
Prior AIDS, yes vs no	1.0 (0.8-1.4), 0.944	1.0 (0.8-1.3), 0.998	**1.6 (1.1-2.5), 0.048**	1.4 (0.9-2.2), 0.179
Prior ARV use, yes vs no	**1.7 (1.4-2.1), <0.001**	**0.7 (0.6-0.8), <0.001**	1.6 (0.9-2.8), 0.116	1.5 (0.8-3.0), 0.195
Regimen, NNRTI vs PI^b^	0.8 (0.7-1.1), 0.181	0.9 (0.8-1.1), 0.517	0.7 (0.3-1.3), 0.250	1.6 (0.8-3.0), 0.165
STI After HIV, yes vs no	**0.8 (0.7-1.0), 0.048**	1.0 (0.9-1.1), 0.820	1.1 (0.7-1.6), 0.699	1.0 (0.6-1.4), 0.806
Hepatitis B, yes vs no	1.1 (0.8-1.4), 0.733	0.9 (0.7-1.2), 0.454	1.1 (0.7-1.9), 0.667	1.2 (0.6-2.1), 0.612
Hepatitis C, yes vs no	1.2 (0.9-1.7), 0.242	1.3 (1.0-1.7), 0.079	1.4 (0.8-2.5), 0.250	**1.9 (1.1-3.3), 0.026**
Hgb, 2 mg/dl	1.0 (0.9-1.1), 0.774	0.9 (0.8-1.0), 0.089	0.8 (0.6-1.0), 0.111	**0.7 (0.6-0.9), 0.009**

Displayed are the hazard ratios, 95% confidence intervals and p values. The analyses are stratified by treatment era and medical treatment facility.

HAART - Highly Active Antiretroviral Therapy; VL - Viral load; CD4 - CD4 count; ARV - Antiretroviral; AA - African American; EA - European American, STI - Sexually Transmitted Infection; Hgb - Hemoglobin; **bold = p < 0.05**

^a^Hazard Ratio of patients able to achieve CD4 cell increase of at least 50% from the baseline CD4 count

^b^Additional categories examined but not displayed: for ethnicity, other vs EA; for rank, others vs officer; for regimen, neither vs PI and both vs PI

## Discussion

In this study, we describe the clinical characteristics and response to HAART among HIV-infected military personnel and beneficiaries initiating treatment over the course of twelve years (1996-2007) with an average follow-up of over 6 years. The NHS is conducted within the military medical system allowing for an evaluation of HAART response in a U.S. clinical setting with free and open access to healthcare and medications.

After stratifying patients into two HAART initiation eras, 1996-2000 (EHE) and 2000 onward (LHE), it was evident that these eras differed significantly for several reasons. First, the large majority of patients starting treatment in the EHE had prior exposure to suboptimal therapy which has been shown to compromise the response to HAART [19-21]. Secondly, more potent regimens were available in the LHE. Additionally, more patients in the EHE had a prior AIDS-defining illness likely impacting response [22,23]. Furthermore, those who survived the pre-HAART era long enough to initiate HAART may have intrinsic host factors which could impact outcomes [24]. Finally, there were significant differences in the timing of HAART initiation between both eras (duration of HIV diagnosis to HAART initiation and baseline CD4 count). This likely reflects differences in treatment guideline recommendations that were followed in each era and the fact that many patients starting HAART in the EHE became infected well before the availability of HAART. Despite the challenges experienced by participants initiating in the EHE, the percent virologically suppressed was around 60% throughout the duration of follow up.

For the LHE patients in this cohort, the virologic and immunologic responses were similar to those reported by randomized clinical trials using a regimen containing either efavirenz or a boosted-PI. A meta-analysis of 20 clinical trials by Gupta et al. described a VS rate of 76% and CD4 change of 176 cells/mL at 48 weeks [25]. The rates we observed were equivalent or slightly higher than these and were sustained for more than 5 years. Limited population and cohort studies in the U.S. have shown variable VS rates at 3 to 8 months of 50-85% and rebound at 3 years of 20-50% [26,27]. Outside the U.S., several cohorts with universal access to healthcare have demonstrated a remarkable response to HAART when compared to cohorts with similar demographics in the U.S.[3,28-31]. The Swiss HIV Cohort Study reported an overall ITT VS rate of 89% and a CD4 increase of 177 cells/mL at 12 months after HAART initiation for ARV naïve patients during this same LHE [32]. In this same analysis, the percentage of patients having a change or discontinuation within the first year of ART for any reason was 44.3-48.8% (varying by era) which is comparable to patients in the NHS.

Although there are drug assistance programs in the U.S. for eligible individuals with HIV/AIDS, the delay before medical care becomes available can postpone HAART initiation, and even the minimal associated costs can be a significant barrier for some patients [33,34]. Co-payments and fees can reduce adherence and have been shown to increase mortality [35-37]. It is important to note, however, that universal access to care and free medications are insufficient to ensure that all patients will achieve treatment success. Joy et al. described a population in Vancouver, Canada that has open access to healthcare but found that poverty, unemployment and a lack of post-secondary education impacted on survival in the HAART era [38-40].

This cohort provided an opportunity to examine the relationship between demographic and clinical factors with outcomes after HAART in a clinical setting that minimized confounding related to access to care and IDU. Previously, we and others have shown associations between both age at HAART initiation [41] and ethnicity [17,42,43] with treatment response. Concordant with other studies, viral load was a predictor of VF and mortality and CD4 count was a predictor of immune reconstitution, AIDS events, and mortality [44-46]. The CD4 recovery was greatest for those with lower baseline CD4 counts similar to findings by Hunt et al. [47] which likely reflects the endpoint used in this analysis (50% increase). We also showed an association between the duration of HIV infection and CD4 reconstitution [48] in addition to increased AIDS events despite a lack of evidence for these findings in a previous prospective study [49]. Although conflicting findings abound in the literature with respect to gender differences in HAART response [50], in the present study, women had a longer time to AIDS as compared to men (consistent with several similar reports [51-54]). Surprisingly, among all subjects the initial regimen type was not found to be a significant predictor of VF [55]. Although this analysis did not distinguish among the NNRTIs or boosted-PIs from unboosted-PIs, the era stratification accounted for differences in drug potency. Interestingly in our study, patients with a prior STI had a lower rate of VF. This is in contrast to studies showing a higher incidence of STIs being associated with non-adherence [56-58] or a negative impact on VL and CD4 count likely via increased immune activation [59].

Active duty status was associated with improved survival, immune reconstitution and a lower rate of AIDS-defining events. Although a distinctive factor in our cohort, important implications related to adherence and general health can be proposed as to why individuals on active duty had improved outcomes; some of which could be translated to other settings. Factors that might improve an active duty member's medication adherence include: (1) better access to ARVs, (2) closer clinical monitoring, and (3) a more disciplined and regimented environment. Although all participants in this cohort study do have free access to the DoD healthcare system, retirees can live further from network facilities and can choose private insurance resulting in copayments for ARVs. Furthermore, active duty personnel may be more closely monitored as they are required by their supervisors to seek medical care on a regular basis. As evidence, research study visit attendance has been shown to be significantly greater for active duty vs. others [17]. General health may be better among active duty members because of physical fitness requirements, lower rates of substance abuse, and a cultural awareness of the benefits of health and nutrition [4,29,60]. Additional factors such as stable employment and guaranteed housing may also contribute to better outcomes. Finally, the goal of remaining on active duty itself is an incentive to stay healthy. HIV-infected military personnel can remain on active duty and continue working, but the development of an AIDS-defining illness can lead to medical separation with retention of health benefits. Although the MV analysis adjusted for several clinical factors such as previous AIDS event, it is possible that non-active duty status is a marker for poorer health. This is substantiated by the fact that 28% of non-active duty patients were retired for medical reasons prior to the start of HAART.

One limitation of this study is that medication adherence data were unavailable for most patients (adherence questionnaires were added to the data collection in 2006). The relative impact of HIV drug resistance was also not assessed in this study. Finally, a disadvantage of any cohort study is that these results cannot be readily extrapolated to other clinical settings where rates of IDU, demographic characteristics, and access to healthcare differ. However, this cohort does provide an opportunity to observe sustainable treatment success after early HAART initiation under these conditions.

## Conclusions

In summary, we find rates of VS and CD4 reconstitution to be high and clinical events to be low for DoD beneficiaries receiving treatment for HIV. These rates approach those reported in clinical trials. Active duty personnel have better immunologic and clinical outcomes but equivalent rates of VF to other beneficiaries. These findings support the notion that free and open access to healthcare provides a favorable environment for optimizing HIV treatment outcomes.

## Competing interests

The authors declare that they have no competing interests.

## Authors' contributions

The following authors were involved in study conception and design: VCM, GG, ACW, BKA; acquisition of data: VCM, ACW, HC, MLL, AG, JFO, NCC, RJO, GWW, BKA; analysis and interpretation of data: VCM, GG, ACW, BKA; manuscript drafting and critical revision: VCM, GG, ACW, MLL, AG, JFO, NCC, RJO, AL, GWW, BKA. All authors read and approved the final manuscript.
